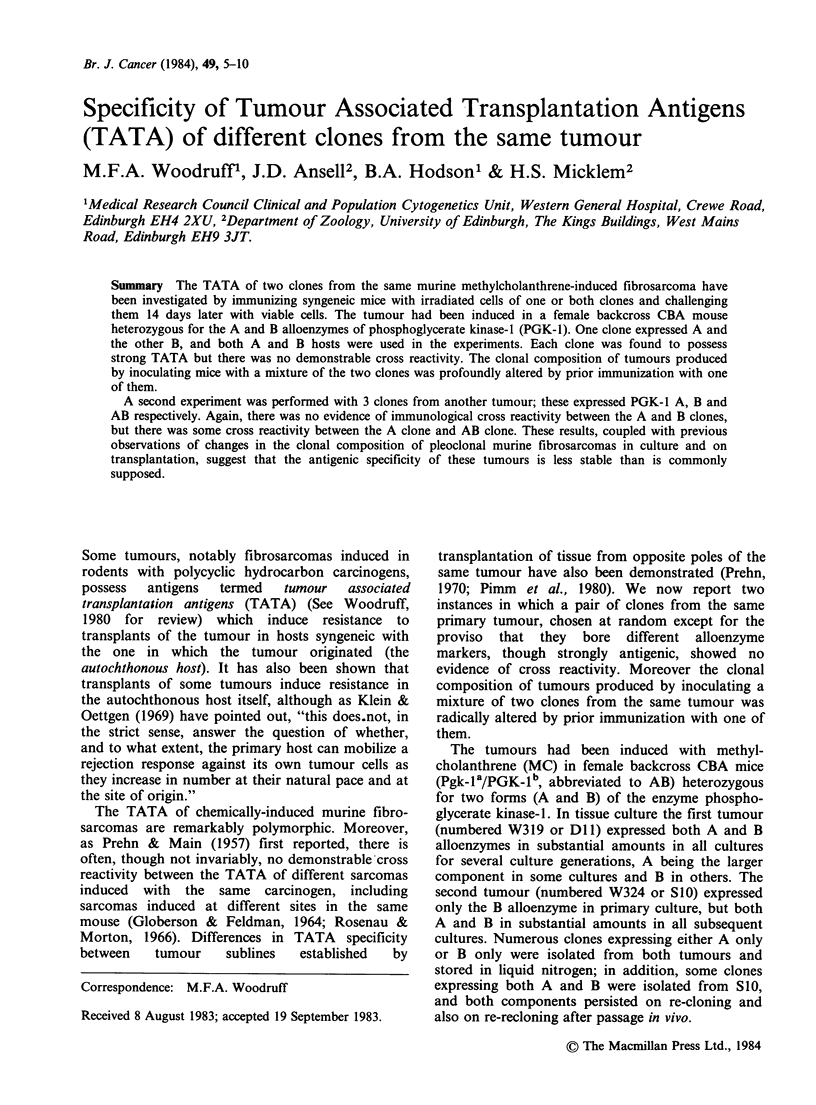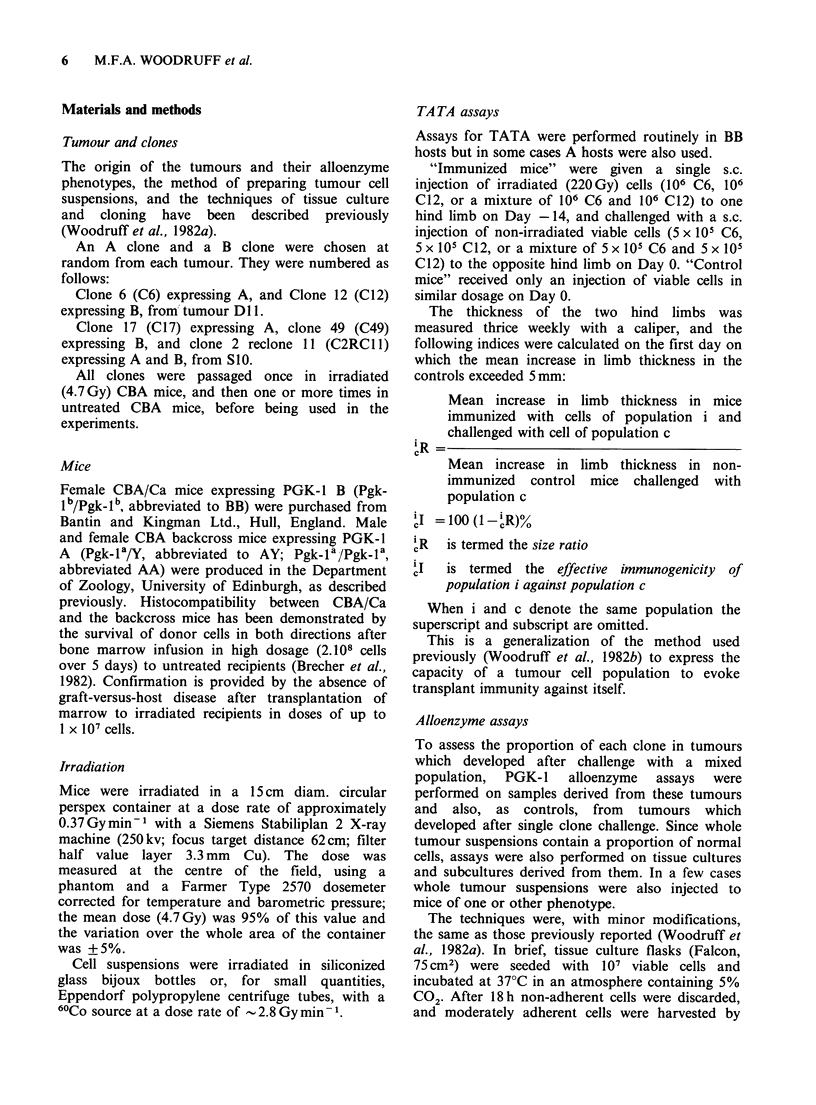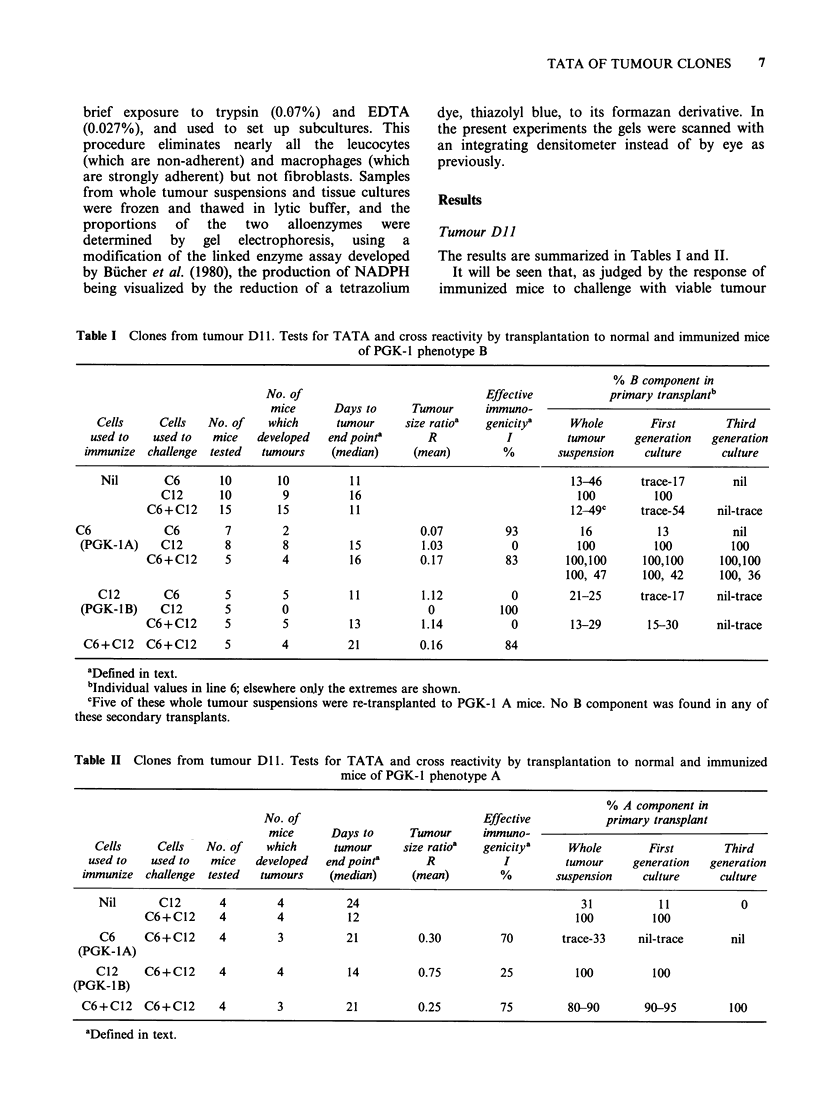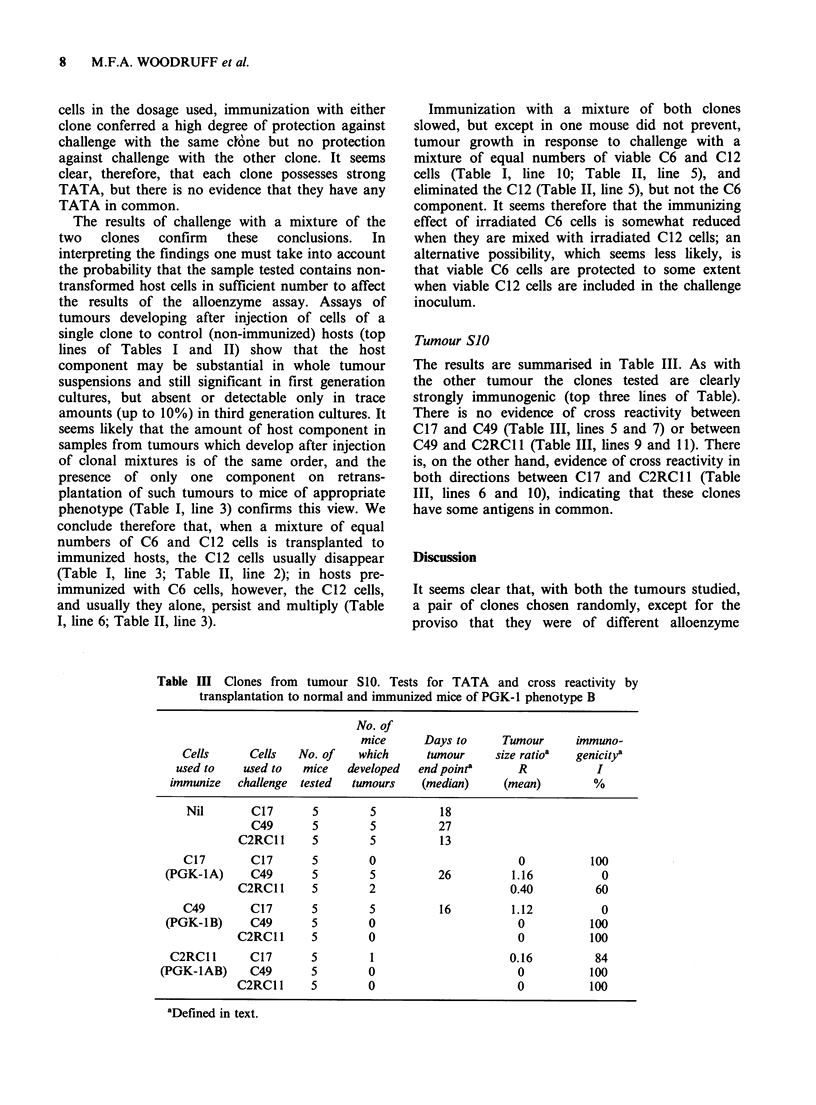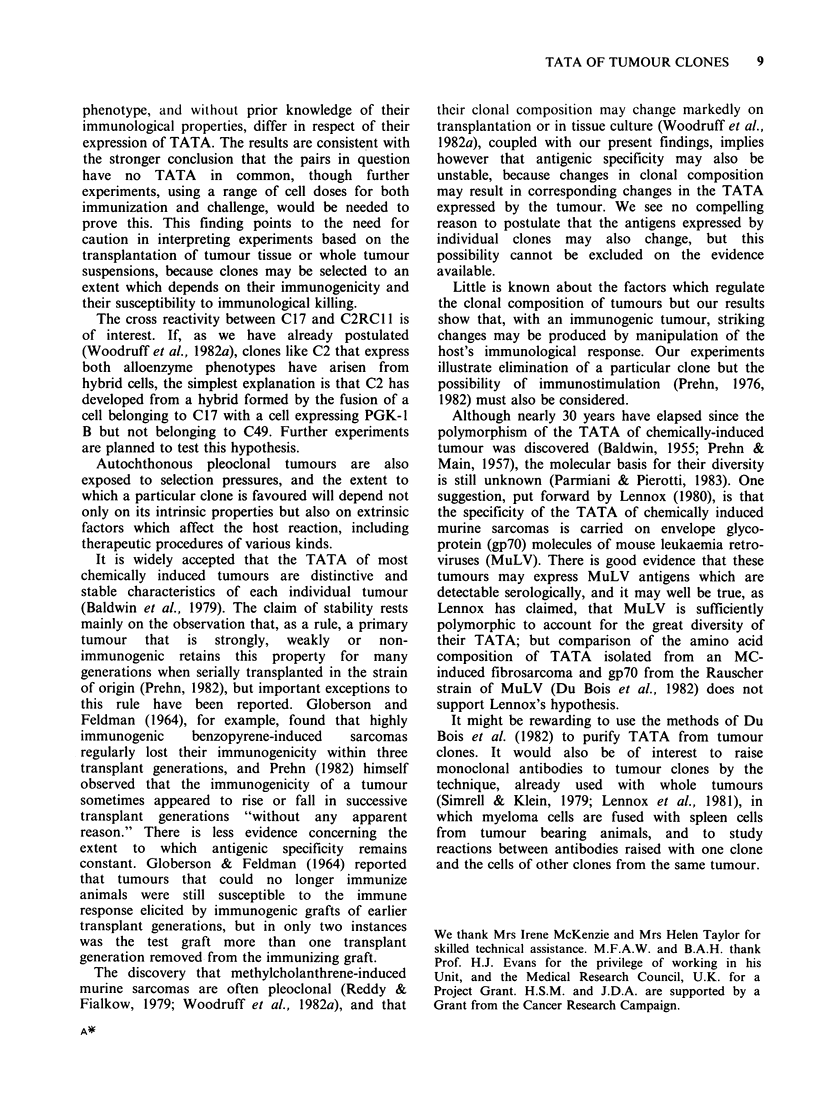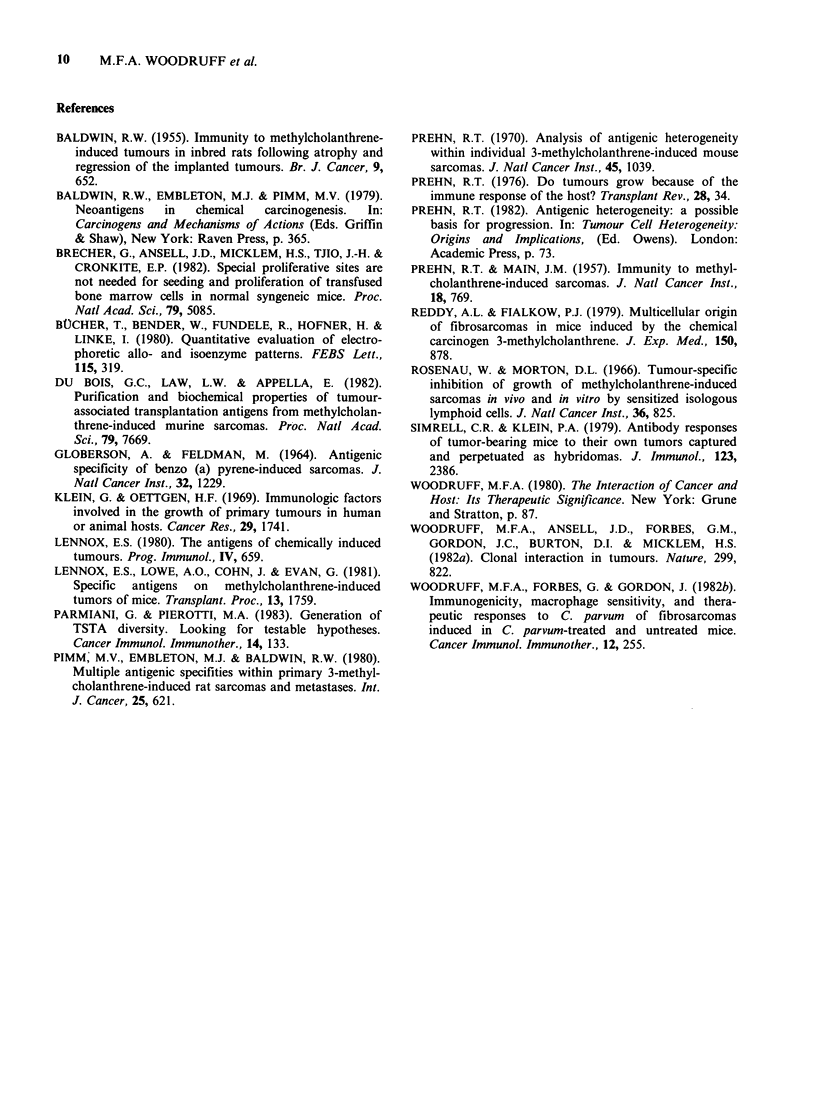# Specificity of tumour associated transplantation antigens (TATA) of different clones from the same tumour.

**DOI:** 10.1038/bjc.1984.2

**Published:** 1984-01

**Authors:** M. F. Woodruff, J. D. Ansell, B. A. Hodson, H. S. Micklem

## Abstract

The TATA of two clones from the same murine methylcholanthrene-induced fibrosarcoma have been investigated by immunizing syngeneic mice with irradiated cells of one or both clones and challenging them 14 days later with viable cells. The tumour had been induced in a female backcross CBA mouse heterozygous for the A and B alloenzymes of phosphoglycerate kinase-1 (PGK-1). One clone expressed A and the other B, and both A and B hosts were used in the experiments. Each clone was found to possess strong TATA but there was no demonstrable cross reactivity. The clonal composition of tumours produced by inoculating mice with a mixture of the two clones was profoundly altered by prior immunization with one of them. A second experiment was performed with 3 clones from another tumour; these expressed PGK-1 A, B and AB respectively. Again, there was no evidence of immunological cross reactivity between the A and B clones, but there was some cross reactivity between the A clone and AB clone. These results, coupled with previous observations of changes in the clonal composition of pleoclonal murine fibrosarcomas in culture and on transplantation, suggest that the antigenic specificity of these tumours is less stable than is commonly supposed.


*Br. J. Cancer* (1984), **49**, 5–10

### Specificity of Tumour Associated Transplantation Antigens (TATA) of different clones from the same tumour

M.F.A. Woodruff[1], J.D. Ansell[2], B.A. Hodson[1] & H.S. Micklem[2]

[1]*Medical Research Council Clinical and Population Cytogenetics Unit, Western General Hospital, Crewe Road, Edinburgh EH4 2XU,* [2]*Department of Zoology, University of Edinburgh, The Kings Buildings, West Mains Road, Edinburgh EH9 3JT.*

**Summary** The TATA of two clones from the same murine methylcholanthrene-induced fibrosarcoma have been investigated by immunizing syngeneic mice with irradiated cells of one or both clones and challenging them 14 days later with viable cells. The tumour had been induced in a female backcross CBA mouse heterozygous for the A and B alloenzymes of phosphoglycerate kinase-1 (PGK-1). One clone expressed A and the other B, and both A and B hosts were used in the experiments. Each clone was found to possess strong TATA but there was no demonstrable cross reactivity. The clonal composition of tumours produced by inoculating mice with a mixture of the two clones was profoundly altered by prior immunization with one of them.

A second experiment was performed with 3 clones from another tumour; these expressed PGK-1 A, B and AB respectively. Again, there was no evidence of immunological cross reactivity between the A and B clones, but there was some cross reactivity between the A clone and AB clone. These results, coupled with previous observations of changes in the clonal composition of pleoclonal murine fibrosarcomas in culture and on transplantation, suggest that the antigenic specificity of these tumours is less stable than is commonly supposed.

Some tumours, notably fibrosarcomas induced in rodents with polycyclic hydrocarbon carcinogens, possess antigens termed *tumour associated transplantation antigens* (TATA) (See Woodruff, 1980 for review) which induce resistance to transplants of the tumour in hosts syngeneic with the one in which the tumour originated (the *autochthonous host*). It has also been shown that transplants of some tumours induce resistance in the autochthonous host itself, although as Klein & Oettgen (1969) have pointed out, "this does.not, in the strict sense, answer the question of whether, and to what extent, the primary host can mobilize a rejection response against its own tumour cells as they increase in number at their natural pace and at the site of origin."

The TATA of chemically-induced murine fibrosarcomas are remarkably polymorphic. Moreover, as Prehn & Main (1957) first reported, there is often, though not invariably, no demonstrable cross reactivity between the TATA of different sarcomas induced with the same carcinogen, including sarcomas induced at different sites in the same mouse (Globerson & Feldman, 1964; Rosenau & Morton, 1966). Differences in TATA specificity between tumour sublines established by transplantation of tissue from opposite poles of the same tumour have also been demonstrated (Prehn, 1970; Pimm *et al.*, 1980). We now report two instances in which a pair of clones from the same primary tumour, chosen at random except for the proviso that they bore different alloenzyme markers, though strongly antigenic, showed no evidence of cross reactivity. Moreover the clonal composition of tumours produced by inoculating a mixture of two clones from the same tumour was radically altered by prior immunization with one of them.

The tumours had been induced with methylcholanthrene (MC) in female backcross CBA mice ($Pgk\text{-}1^a/PGK\text{-}1^b$, abbreviated to AB) heterozygous for two forms (A and B) of the enzyme phosphoglycerate kinase-1. In tissue culture the first tumour (numbered W319 or D11) expressed both A and B alloenzymes in substantial amounts in all cultures for several culture generations, A being the larger component in some cultures and B in others. The second tumour (numbered W324 or S10) expressed only the B alloenzyme in primary culture, but both A and B in substantial amounts in all subsequent cultures. Numerous clones expressing either A only or B only were isolated from both tumours and stored in liquid nitrogen; in addition, some clones expressing both A and B were isolated from S10, and both components persisted on re-cloning and also on re-recloning after passage *in vivo*.

Correspondence: M.F.A. Woodruff

Received 8 August 1983; accepted 19 September 1983.

© The Macmillan Press Ltd., 1984

### Materials and methods

*Tumour and clones*

The origin of the tumours and their alloenzyme phenotypes, the method of preparing tumour cell suspensions, and the techniques of tissue culture and cloning have been described previously (Woodruff *et al.*, 1982*a*).

An A clone and a B clone were chosen at random from each tumour. They were numbered as follows:

Clone 6 (C6) expressing A, and Clone 12 (C12) expressing B, from tumour D11.

Clone 17 (C17) expressing A, clone 49 (C49) expressing B, and clone 2 reclone 11 (C2RC11) expressing A and B, from S10.

All clones were passaged once in irradiated (4.7 Gy) CBA mice, and then one or more times in untreated CBA mice, before being used in the experiments.

*Mice*

Female CBA/Ca mice expressing PGK-1 B (Pgk-$1^b$/Pgk-$1^b$, abbreviated to BB) were purchased from Bantin and Kingman Ltd., Hull, England. Male and female CBA backcross mice expressing PGK-1 A (Pgk-$1^a$/Y, abbreviated to AY; Pgk-$1^a$/Pgk-$1^a$, abbreviated AA) were produced in the Department of Zoology, University of Edinburgh, as described previously. Histocompatibility between CBA/Ca and the backcross mice has been demonstrated by the survival of donor cells in both directions after bone marrow infusion in high dosage ($2.10^8$ cells over 5 days) to untreated recipients (Brecher *et al.*, 1982). Confirmation is provided by the absence of graft-versus-host disease after transplantation of marrow to irradiated recipients in doses of up to $1 \times 10^7$ cells.

*Irradiation*

Mice were irradiated in a 15 cm diam. circular perspex container at a dose rate of approximately $0.37\,Gy\,min^{-1}$ with a Siemens Stabiliplan 2 X-ray machine (250 kv; focus target distance 62 cm; filter half value layer 3.3 mm Cu). The dose was measured at the centre of the field, using a phantom and a Farmer Type 2570 dosemeter corrected for temperature and barometric pressure; the mean dose (4.7 Gy) was 95% of this value and the variation over the whole area of the container was $\pm 5\%$.

Cell suspensions were irradiated in siliconized glass bijoux bottles or, for small quantities, Eppendorf polypropylene centrifuge tubes, with a $^{60}Co$ source at a dose rate of $\sim 2.8\,Gy\,min^{-1}$.

*TATA assays*

Assays for TATA were performed routinely in BB hosts but in some cases A hosts were also used.

"Immunized mice" were given a single s.c. injection of irradiated (220 Gy) cells ($10^6$ C6, $10^6$ C12, or a mixture of $10^6$ C6 and $10^6$ C12) to one hind limb on Day −14, and challenged with a s.c. injection of non-irradiated viable cells ($5 \times 10^5$ C6, $5 \times 10^5$ C12, or a mixture of $5 \times 10^5$ C6 and $5 \times 10^5$ C12) to the opposite hind limb on Day 0. "Control mice" received only an injection of viable cells in similar dosage on Day 0.

The thickness of the two hind limbs was measured thrice weekly with a caliper, and the following indices were calculated on the first day on which the mean increase in limb thickness in the controls exceeded 5 mm:

$$ {}^{i}_{c}R = \frac{\text{Mean increase in limb thickness in mice immunized with cells of population i and challenged with cell of population c}}{\text{Mean increase in limb thickness in non-immunized control mice challenged with population c}} $$

$$ {}^{i}_{c}I = 100\,(1 - {}^{i}_{c}R)\% $$

${}^{i}_{c}R$ is termed the *size ratio*

${}^{i}_{c}I$ is termed the *effective immunogenicity of population i against population c*

When i and c denote the same population the superscript and subscript are omitted.

This is a generalization of the method used previously (Woodruff *et al.*, 1982*b*) to express the capacity of a tumour cell population to evoke transplant immunity against itself.

*Alloenzyme assays*

To assess the proportion of each clone in tumours which developed after challenge with a mixed population, PGK-1 alloenzyme assays were performed on samples derived from these tumours and also, as controls, from tumours which developed after single clone challenge. Since whole tumour suspensions contain a proportion of normal cells, assays were also performed on tissue cultures and subcultures derived from them. In a few cases whole tumour suspensions were also injected to mice of one or other phenotype.

The techniques were, with minor modifications, the same as those previously reported (Woodruff *et al.*, 1982*a*). In brief, tissue culture flasks (Falcon, $75\,cm^2$) were seeded with $10^7$ viable cells and incubated at 37°C in an atmosphere containing 5% $CO_2$. After 18 h non-adherent cells were discarded, and moderately adherent cells were harvested by brief exposure to trypsin (0.07%) and EDTA (0.027%), and used to set up subcultures. This procedure eliminates nearly all the leucocytes (which are non-adherent) and macrophages (which are strongly adherent) but not fibroblasts. Samples from whole tumour suspensions and tissue cultures were frozen and thawed in lytic buffer, and the proportions of the two alloenzymes were determined by gel electrophoresis, using a modification of the linked enzyme assay developed by Bücher *et al.* (1980), the production of NADPH being visualized by the reduction of a tetrazolium dye, thiazolyl blue, to its formazan derivative. In the present experiments the gels were scanned with an integrating densitometer instead of by eye as previously.

### Results

### *Tumour D11*

The results are summarized in Tables I and II.

It will be seen that, as judged by the response of immunized mice to challenge with viable tumour

**Table I** Clones from tumour D11. Tests for TATA and cross reactivity by transplantation to normal and immunized mice of PGK-1 phenotype B

| *Cells used to immunize* | *Cells used to challenge* | *No. of mice tested* | *No. of mice which developed tumours* | *Days to tumour end point*[a] *(median)* | *Tumour size ratio*[a] *R (mean)* | *Effective immuno-genicity*[a] *I %* | *% B component in primary transplant*[b] | | |
|---|---|---|---|---|---|---|---|---|---|
| | | | | | | | *Whole tumour suspension* | *First generation culture* | *Third generation culture* |
| Nil | C6 | 10 | 10 | 11 | | | 13–46 | trace-17 | nil |
| | C12 | 10 | 9 | 16 | | | 100 | 100 | |
| | C6+C12 | 15 | 15 | 11 | | | 12–49[c] | trace-54 | nil-trace |
| C6 (PGK-1A) | C6 | 7 | 2 | | 0.07 | 93 | 16 | 13 | nil |
| | C12 | 8 | 8 | 15 | 1.03 | 0 | 100 | 100 | 100 |
| | C6+C12 | 5 | 4 | 16 | 0.17 | 83 | 100,100<br>100, 47 | 100,100<br>100, 42 | 100,100<br>100, 36 |
| C12 (PGK-1B) | C6 | 5 | 5 | 11 | 1.12 | 0 | 21–25 | trace-17 | nil-trace |
| | C12 | 5 | 0 | | 0 | 100 | | | |
| | C6+C12 | 5 | 5 | 13 | 1.14 | 0 | 13–29 | 15–30 | nil-trace |
| C6+C12 | C6+C12 | 5 | 4 | 21 | 0.16 | 84 | | | |

[a]Defined in text.
[b]Individual values in line 6; elsewhere only the extremes are shown.
[c]Five of these whole tumour suspensions were re-transplanted to PGK-1 A mice. No B component was found in any of these secondary transplants.

**Table II** Clones from tumour D11. Tests for TATA and cross reactivity by transplantation to normal and immunized mice of PGK-1 phenotype A

| *Cells used to immunize* | *Cells used to challenge* | *No. of mice tested* | *No. of mice which developed tumours* | *Days to tumour end point*[a] *(median)* | *Tumour size ratio*[a] *R (mean)* | *Effective immuno-genicity*[a] *I %* | *% A component in primary transplant* | | |
|---|---|---|---|---|---|---|---|---|---|
| | | | | | | | *Whole tumour suspension* | *First generation culture* | *Third generation culture* |
| Nil | C12 | 4 | 4 | 24 | | | 31 | 11 | 0 |
| | C6+C12 | 4 | 4 | 12 | | | 100 | 100 | |
| C6 (PGK-1A) | C6+C12 | 4 | 3 | 21 | 0.30 | 70 | trace-33 | nil-trace | nil |
| C12 (PGK-1B) | C6+C12 | 4 | 4 | 14 | 0.75 | 25 | 100 | 100 | |
| C6+C12 | C6+C12 | 4 | 3 | 21 | 0.25 | 75 | 80–90 | 90–95 | 100 |

[a]Defined in text.

cells in the dosage used, immunization with either clone conferred a high degree of protection against challenge with the same clone but no protection against challenge with the other clone. It seems clear, therefore, that each clone possesses strong TATA, but there is no evidence that they have any TATA in common.

The results of challenge with a mixture of the two clones confirm these conclusions. In interpreting the findings one must take into account the probability that the sample tested contains non-transformed host cells in sufficient number to affect the results of the alloenzyme assay. Assays of tumours developing after injection of cells of a single clone to control (non-immunized) hosts (top lines of Tables I and II) show that the host component may be substantial in whole tumour suspensions and still significant in first generation cultures, but absent or detectable only in trace amounts (up to 10%) in third generation cultures. It seems likely that the amount of host component in samples from tumours which develop after injection of clonal mixtures is of the same order, and the presence of only one component on retransplantation of such tumours to mice of appropriate phenotype (Table I, line 3) confirms this view. We conclude therefore that, when a mixture of equal numbers of C6 and C12 cells is transplanted to immunized hosts, the C12 cells usually disappear (Table I, line 3; Table II, line 2); in hosts pre-immunized with C6 cells, however, the C12 cells, and usually they alone, persist and multiply (Table I, line 6; Table II, line 3).

Immunization with a mixture of both clones slowed, but except in one mouse did not prevent, tumour growth in response to challenge with a mixture of equal numbers of viable C6 and C12 cells (Table I, line 10; Table II, line 5), and eliminated the C12 (Table II, line 5), but not the C6 component. It seems therefore that the immunizing effect of irradiated C6 cells is somewhat reduced when they are mixed with irradiated C12 cells; an alternative possibility, which seems less likely, is that viable C6 cells are protected to some extent when viable C12 cells are included in the challenge inoculum.

### *Tumour S10*

The results are summarised in Table III. As with the other tumour the clones tested are clearly strongly immunogenic (top three lines of Table). There is no evidence of cross reactivity between C17 and C49 (Table III, lines 5 and 7) or between C49 and C2RC11 (Table III, lines 9 and 11). There is, on the other hand, evidence of cross reactivity in both directions between C17 and C2RC11 (Table III, lines 6 and 10), indicating that these clones have some antigens in common.

### Discussion

It seems clear that, with both the tumours studied, a pair of clones chosen randomly, except for the proviso that they were of different alloenzyme

**Table III** Clones from tumour S10. Tests for TATA and cross reactivity by transplantation to normal and immunized mice of PGK-1 phenotype B

| *Cells used to immunize* | *Cells used to challenge* | *No. of mice tested* | *No. of mice which developed tumours* | *Days to tumour end point*[a] *(median)* | *Tumour size ratio*[a] *R (mean)* | *immuno-genicity*[a] *I* % |
|---|---|---|---|---|---|---|
| Nil | C17 | 5 | 5 | 18 | | |
| | C49 | 5 | 5 | 27 | | |
| | C2RC11 | 5 | 5 | 13 | | |
| C17 (PGK-1A) | C17 | 5 | 0 | | 0 | 100 |
| | C49 | 5 | 5 | 26 | 1.16 | 0 |
| | C2RC11 | 5 | 2 | | 0.40 | 60 |
| C49 (PGK-1B) | C17 | 5 | 5 | 16 | 1.12 | 0 |
| | C49 | 5 | 0 | | 0 | 100 |
| | C2RC11 | 5 | 0 | | 0 | 100 |
| C2RC11 (PGK-1AB) | C17 | 5 | 1 | | 0.16 | 84 |
| | C49 | 5 | 0 | | 0 | 100 |
| | C2RC11 | 5 | 0 | | 0 | 100 |

[a]Defined in text.

phenotype, and without prior knowledge of their immunological properties, differ in respect of their expression of TATA. The results are consistent with the stronger conclusion that the pairs in question have no TATA in common, though further experiments, using a range of cell doses for both immunization and challenge, would be needed to prove this. This finding points to the need for caution in interpreting experiments based on the transplantation of tumour tissue or whole tumour suspensions, because clones may be selected to an extent which depends on their immunogenicity and their susceptibility to immunological killing.

The cross reactivity between C17 and C2RC11 is of interest. If, as we have already postulated (Woodruff *et al.*, 1982*a*), clones like C2 that express both alloenzyme phenotypes have arisen from hybrid cells, the simplest explanation is that C2 has developed from a hybrid formed by the fusion of a cell belonging to C17 with a cell expressing PGK-1 B but not belonging to C49. Further experiments are planned to test this hypothesis.

Autochthonous pleoclonal tumours are also exposed to selection pressures, and the extent to which a particular clone is favoured will depend not only on its intrinsic properties but also on extrinsic factors which affect the host reaction, including therapeutic procedures of various kinds.

It is widely accepted that the TATA of most chemically induced tumours are distinctive and stable characteristics of each individual tumour (Baldwin *et al.*, 1979). The claim of stability rests mainly on the observation that, as a rule, a primary tumour that is strongly, weakly or non-immunogenic retains this property for many generations when serially transplanted in the strain of origin (Prehn, 1982), but important exceptions to this rule have been reported. Globerson and Feldman (1964), for example, found that highly immunogenic benzopyrene-induced sarcomas regularly lost their immunogenicity within three transplant generations, and Prehn (1982) himself observed that the immunogenicity of a tumour sometimes appeared to rise or fall in successive transplant generations "without any apparent reason." There is less evidence concerning the extent to which antigenic specificity remains constant. Globerson & Feldman (1964) reported that tumours that could no longer immunize animals were still susceptible to the immune response elicited by immunogenic grafts of earlier transplant generations, but in only two instances was the test graft more than one transplant generation removed from the immunizing graft.

The discovery that methylcholanthrene-induced murine sarcomas are often pleoclonal (Reddy & Fialkow, 1979; Woodruff *et al.*, 1982*a*), and that their clonal composition may change markedly on transplantation or in tissue culture (Woodruff *et al.*, 1982*a*), coupled with our present findings, implies however that antigenic specificity may also be unstable, because changes in clonal composition may result in corresponding changes in the TATA expressed by the tumour. We see no compelling reason to postulate that the antigens expressed by individual clones may also change, but this possibility cannot be excluded on the evidence available.

Little is known about the factors which regulate the clonal composition of tumours but our results show that, with an immunogenic tumour, striking changes may be produced by manipulation of the host's immunological response. Our experiments illustrate elimination of a particular clone but the possibility of immunostimulation (Prehn, 1976, 1982) must also be considered.

Although nearly 30 years have elapsed since the polymorphism of the TATA of chemically-induced tumour was discovered (Baldwin, 1955; Prehn & Main, 1957), the molecular basis for their diversity is still unknown (Parmiani & Pierotti, 1983). One suggestion, put forward by Lennox (1980), is that the specificity of the TATA of chemically induced murine sarcomas is carried on envelope glycoprotein (gp70) molecules of mouse leukaemia retroviruses (MuLV). There is good evidence that these tumours may express MuLV antigens which are detectable serologically, and it may well be true, as Lennox has claimed, that MuLV is sufficiently polymorphic to account for the great diversity of their TATA; but comparison of the amino acid composition of TATA isolated from an MC-induced fibrosarcoma and gp70 from the Rauscher strain of MuLV (Du Bois *et al.*, 1982) does not support Lennox's hypothesis.

It might be rewarding to use the methods of Du Bois *et al.* (1982) to purify TATA from tumour clones. It would also be of interest to raise monoclonal antibodies to tumour clones by the technique, already used with whole tumours (Simrell & Klein, 1979; Lennox *et al.*, 1981), in which myeloma cells are fused with spleen cells from tumour bearing animals, and to study reactions between antibodies raised with one clone and the cells of other clones from the same tumour.

We thank Mrs Irene McKenzie and Mrs Helen Taylor for skilled technical assistance. M.F.A.W. and B.A.H. thank Prof. H.J. Evans for the privilege of working in his Unit, and the Medical Research Council, U.K. for a Project Grant. H.S.M. and J.D.A. are supported by a Grant from the Cancer Research Campaign.

### References

BALDWIN, R.W. (1955). Immunity to methylcholanthrene-induced tumours in inbred rats following atrophy and regression of the implanted tumours. *Br. J. Cancer*, **9**, 652.

BALDWIN, R.W., EMBLETON, M.J. & PIMM, M.V. (1979). Neoantigens in chemical carcinogenesis. In: *Carcinogens and Mechanisms of Actions* (Eds. Griffin & Shaw), New York: Raven Press, p. 365.

BRECHER, G., ANSELL, J.D., MICKLEM, H.S., TJIO, J.-H. & CRONKITE, E.P. (1982). Special proliferative sites are not needed for seeding and proliferation of transfused bone marrow cells in normal syngeneic mice. *Proc. Natl Acad. Sci.*, **79**, 5085.

BÜCHER, T., BENDER, W., FUNDELE, R., HOFNER, H. & LINKE, I. (1980). Quantitative evaluation of electrophoretic allo- and isoenzyme patterns. *FEBS Lett.*, **115**, 319.

DU BOIS, G.C., LAW, L.W. & APPELLA, E. (1982). Purification and biochemical properties of tumour-associated transplantation antigens from methylcholanthrene-induced murine sarcomas. *Proc. Natl Acad. Sci.*, **79**, 7669.

GLOBERSON, A. & FELDMAN, M. (1964). Antigenic specificity of benzo (a) pyrene-induced sarcomas. *J. Natl Cancer Inst.*, **32**, 1229.

KLEIN, G. & OETTGEN, H.F. (1969). Immunologic factors involved in the growth of primary tumours in human or animal hosts. *Cancer Res.*, **29**, 1741.

LENNOX, E.S. (1980). The antigens of chemically induced tumours. *Prog. Immunol.*, **IV**, 659.

LENNOX, E.S., LOWE, A.O., COHN, J. & EVAN, G. (1981). Specific antigens on methylcholanthrene-induced tumors of mice. *Transplant. Proc.*, **13**, 1759.

PARMIANI, G. & PIEROTTI, M.A. (1983). Generation of TSTA diversity. Looking for testable hypotheses. *Cancer Immunol. Immunother.*, **14**, 133.

PIMM, M.V., EMBLETON, M.J. & BALDWIN, R.W. (1980). Multiple antigenic specifities within primary 3-methylcholanthrene-induced rat sarcomas and metastases. *Int. J. Cancer*, **25**, 621.

PREHN, R.T. (1970). Analysis of antigenic heterogeneity within individual 3-methylcholanthrene-induced mouse sarcomas. *J. Natl Cancer Inst.*, **45**, 1039.

PREHN, R.T. (1976). Do tumours grow because of the immune response of the host? *Transplant Rev.*, **28**, 34.

PREHN, R.T. (1982). Antigenic heterogeneity: a possible basis for progression. In: *Tumour Cell Heterogeneity: Origins and Implications*, (Ed. Owens). London: Academic Press, p. 73.

PREHN, R.T. & MAIN, J.M. (1957). Immunity to methylcholanthrene-induced sarcomas. *J. Natl Cancer Inst.*, **18**, 769.

REDDY, A.L. & FIALKOW, P.J. (1979). Multicellular origin of fibrosarcomas in mice induced by the chemical carcinogen 3-methylcholanthrene. *J. Exp. Med.*, **150**, 878.

ROSENAU, W. & MORTON, D.L. (1966). Tumour-specific inhibition of growth of methylcholanthrene-induced sarcomas *in vivo* and *in vitro* by sensitized isologous lymphoid cells. *J. Natl Cancer Inst.*, **36**, 825.

SIMRELL, C.R. & KLEIN, P.A. (1979). Antibody responses of tumor-bearing mice to their own tumors captured and perpetuated as hybridomas. *J. Immunol.*, **123**, 2386.

WOODRUFF, M.F.A. (1980). *The Interaction of Cancer and Host: Its Therapeutic Significance*. New York: Grune and Stratton, p. 87.

WOODRUFF, M.F.A., ANSELL, J.D., FORBES, G.M., GORDON, J.C., BURTON, D.I. & MICKLEM, H.S. (1982*a*). Clonal interaction in tumours. *Nature*, 299, 822.

WOODRUFF, M.F.A., FORBES, G. & GORDON, J. (1982*b*). Immunogenicity, macrophage sensitivity, and therapeutic responses to *C. parvum* of fibrosarcomas induced in *C. parvum*-treated and untreated mice. *Cancer Immunol. Immunother.*, **12**, 255.