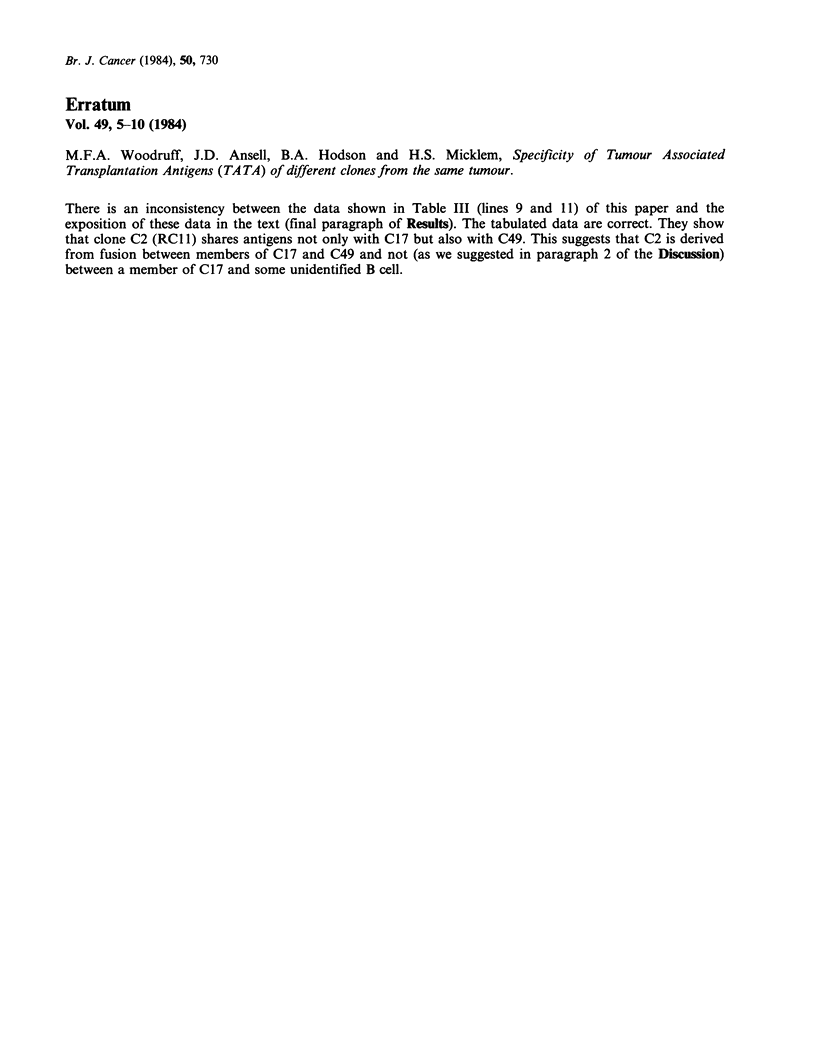# Erratum

**Published:** 1984-11

**Authors:** 


*Br. J. Cancer* (1984), **50**, 730

### Erratum

**Vol. 49, 5–10 (1984)**

M.F.A. Woodruff, J.D. Ansell, B.A. Hodson and H.S. Micklem, *Specificity of Tumour Associated Transplantation Antigens (TATA) of different clones from the same tumour.*

There is an inconsistency between the data shown in Table III (lines 9 and 11) of this paper and the exposition of these data in the text (final paragraph of **Results**). The tabulated data are correct. They show that clone C2 (RC11) shares antigens not only with C17 but also with C49. This suggests that C2 is derived from fusion between members of C17 and C49 and not (as we suggested in paragraph 2 of the **Discussion**) between a member of C17 and some unidentified B cell.